# Differentiations in Gene Content and Expression Response to Virulence Induction Between Two *Agrobacterium* Strains

**DOI:** 10.3389/fmicb.2019.01554

**Published:** 2019-07-09

**Authors:** Mindia Haryono, Shu-Ting Cho, Mei-Jane Fang, Ai-Ping Chen, Shu-Jen Chou, Erh-Min Lai, Chih-Horng Kuo

**Affiliations:** Institute of Plant and Microbial Biology, Academia Sinica, Taipei, Taiwan

**Keywords:** *Agrobacterium*, transformation, virulence, genomics, transcriptomics, RNA-Seq

## Abstract

*Agrobacterium tumefaciens* is important in biotechnology due to its ability to transform eukaryotic cells. Although the molecular mechanisms have been studied extensively, previous studies were focused on the model strain C58. Consequently, nearly all of the commonly used strains for biotechnology application were derived from C58 and share similar host ranges. To overcome this limitation, better understanding of the natural genetic variation could provide valuable insights. In this study, we conducted comparative analysis between C58 and 1D1609. These two strains belong to different genomospecies within the species complex and have distinct infectivity profiles. Genome comparisons revealed that each strain has >1,000 unique genes in addition to the 4,115 shared genes. Furthermore, the divergence in gene content and sequences vary among replicons. The circular chromosome is much more conserved compared to the linear chromosome. To identify the genes that may contribute to their differentiation in virulence, we compared the transcriptomes to screen for genes differentially expressed in response to the inducer acetosyringone. Based on the RNA-Seq results with three biological replicates, ∼100 differentially expressed genes were identified in each strain. Intriguingly, homologous genes with the same expression pattern account for <50% of these differentially expressed genes. This finding indicated that phenotypic variation may be partially explained by divergence in expression regulation. In summary, this study characterized the genomic and transcriptomic differences between two representative *Agrobacterium* strains. Moreover, the short list of differentially expressed genes are promising candidates for future characterization, which could improve our understanding of the genetic mechanisms for phenotypic divergence.

## Introduction

*Agrobacterium tumefaciens* is a natural genetic engineer of its plant hosts (Nester, 2015). During the infection process, a specific segment of DNA originated from its tumor-inducing (Ti) plasmid is transferred to the plant nuclear genome. The genes encoded on this segment of transfer DNA (i.e., T-DNA) include those involved in the biosynthesis of plant hormones (e.g., auxin and cytokinin) and opines (e.g., napoline or octopine). The expression of these genes induces tumor-like cell proliferation on the host plant, a condition that is known as crown gall (Kado, 2014). Moreover, the bacteria residing within the gall could utilize the plant-derived opines as a major source of carbon and nitrogen (Dessaux et al., 1998).

Due to this ability of inter-kingdom DNA transfer, *A. tumefaciens* has been developed into an important tool for genetic manipulation of plants (Hwang et al., 2017). However, despite the multiple advantages of this method, such as the low cost and simple operation, minimal DNA rearrangement, low copy number and high stability of the transferred gene, one major limitation is that many plant species and cultivars have remained difficult to be transformed by the commonly used strains of *A. tumefaciens*. Given this limitation, it is worth noting that all except for one of the ∼15 commonly used strains were derived from C58 (Hellens et al., 2000; Lee and Gelvin, 2008; Hiei et al., 2014), which is the model strain for *Agrobacterium* research (Kado, 2014). Although modifications of the transformation protocols (Wu et al., 2014b) or engineering of those C58-derived strains harboring different types of Ti plasmids (Wu et al., 2014a; Hwang et al., 2015) may further improve the transformation efficiency or host range, a better understanding of the phenotypic and genetic diversity among wild-type *Agrobacterium* strains could provide powerful complementary approaches.

In this regard, extensive variation has been observed within the *A. tumefaciens* species complex (Costechareyre et al., 2009; Costechareyre et al., 2010) in terms of chromosomal background (Lassalle et al., 2011), Ti plasmid type (Hwang et al., 2013), and host range (Hwang et al., 2013). As a part of our effort in investigating the genomic diversity of *A. tumefaciens*, recently we determined the complete genome sequence of a wild-type strain, 1D1609 (Cho et al., 2018). This strain was initially isolated from alfalfa (Palumbo et al., 1998), belongs to the genomospecies 7 (G7) within the *A. tumefaciens* species complex (Cho et al., 2018), and harbors an octopine-type Ti plasmid (Hwang et al., 2013; Cho et al., 2018). Compared to the model strain C58, which was isolated from cherry (Lin and Kado, 1977), belongs to the genomospecies 8 (G8) (Costechareyre et al., 2010), and harbors a nopaline-type Ti plasmid (Goodner et al., 2001; Wood et al., 2001; Slater et al., 2013; Hwang et al., 2013), 1D1609 has lower infection efficiencies against Brassicaceae hosts but higher efficiencies against Leguminosae hosts (Hwang et al., 2013). To establish the links between phenotype and genotype, we compared the genome organization between these two strains, as well as investigated their gene expression response to virulence gene induction in this study.

## Materials and Methods

### Comparative Genomics

All bioinformatic tools were used with the default settings unless stated otherwise. The complete genome sequences of the two strains used in this study, C58 (Goodner et al., 2001; Wood et al., 2001; Slater et al., 2013) and 1D1609 (Cho et al., 2018), were obtained from GenBank (Benson et al., 2018) (accessions: C58, AE007869-AE007872; 1D1609, CP026924-CP026928). The procedures for comparative genomics analysis were based on those described in our previous studies (Chung et al., 2013; Lo et al., 2013). Briefly, the homologous gene clusters were identified using OrthoMCL (Li et al., 2003). The protein sequences of conserved single-copy genes were aligned using MUSCLE v3.8 (Edgar, 2004), concatenated into a single alignment, and used to calculate average amino acid sequence similarity using PHYLIP v3.69 (Felsenstein, 1989). The genome alignment was performed using MAUVE (Darling et al., 2004). For functional category assignments, all protein-coding genes were processed using the BlastKOALA tool (Kanehisa et al., 2016) to identify the corresponding KEGG Orthology numbers, which were then mapped to the COG (Tatusov et al., 2003) functional categories.

### Virulence Gene Induction

To investigate the gene expression regulation, we adopted the protocol described in a previous study (Lee et al., 2013) that uses a chemical inducer acetosyringone to mimic the virulence condition. The gene expression under the induced condition (i.e., in the presence of acetosyringone) was compared to the control condition (i.e., in the absence of acetosyringone) for the identification of differentially expressed genes.

Briefly, each strain was grown at 28°C for 3 days on medium 523 agar plates [10 g sucrose, 8 g casein enzymatic hydrolysate, 4 g yeast extract, 3 g K_2_HPO_4_, 0.3 g MgSO_4_.7H_2_O, 15 g agar per L, pH 7.0]. Seven colonies were individually picked and inoculated in five ml 523 broth at 28°C for 14 h on a shaker incubator (250 rpm). Among the seven samples, three with the closest OD_600_ reading values (i.e., ∼3.2–3.9 for C58 and ∼5.6–6.0 for 1D1609 before 10× dilution for measurement) were selected as the biological replicates and centrifuged at 6,000 × *g* for 4 min at room temperature to collect the cells. The pellet was re-suspended in AB medium [1 g NH_4_Cl, 0.3 g MgSO4.7H_2_O, 0.15 g KCl, 0.01 g CaCl_2_, 2.5 mg FeSO_4_.7H_2_O, 3 g K_2_HPO_4_, 1 g NaH_2_PO_4_, 50 mM 2-(4-morpholinoo)-ethane sulfonic acid (MES), 2% glucose per L, pH 5.5] and adjusted to the equivalent of OD_600_ 10. For each sample, 100 ul of the re-suspended cells was transferred to two sterile tube with 4.9 ml AB medium each, one to be used as the control and the other as the induced set. Both tubes were incubated at 28°C for 6 h on a shaker incubator (250 rpm) before induction. For induction, 5 ul of the virulence gene inducer acetosyringone (200 mM in DMSO) was added to the induced sample and 5 ul of DMSO was added to the control sample. The samples were incubated at 28°C for 16 h on a shaker incubator (250 rpm) prior to RNA extraction using the MasterPure RNA Purification Kit (Epicenter, United States). With three biological replicates for each strain-condition combination, the experiment has a total of 12 samples. To confirm the successful induction of virulence genes, we checked the expression level of three virulence regulon genes (i.e., *virB1*, *virB11*, and *virD2*) by qRT-PCR; *dnaE* was used as the internal control.

### Comparative Transcriptomics

The procedures for comparative transcriptomics were based on those described in our previous study (Lo and Kuo, 2017). Briefly, the strand-specific RNA sequencing (RNA-Seq) library preparation was processed by the core facility of Academia Sinica (Taipei, Taiwan). For each sample, 5 ug of the purified total RNA was used as the starting material. The ribosomal RNA was depleted using the Ribo-Zero rRNA Removal Kit for Bacteria (Cat. No. MRZMB126; Illumina, United States) following the manufacturer’s instructions. Subsequently, the TruSeq Stranded mRNA Library Prep Kit (Cat. No. RS-122-2101; Illumina, United States) was used for sequencing library preparation as described below. The mRNA purification step, which uses the oligo-dT beads to capture polyA tails of eukaryotic mRNA, was skipped because this step is not necessary for bacterial mRNA samples. The rRNA-depleted RNA was fragmented and the first-strand cDNA was synthesized using SuperScript III reverse transcriptase (Cat. No. 18080-093; Invitrogen, United States) with dNTP and random primers according to the manufacturer’s instructions. The second-strand cDNA was generated using a dUTP mix. The double-stranded cDNA was subject to the addition of a single “A” base to the 3′-end, followed by ligation of the barcoded TruSeq adapter. The products were purified and amplified with 10 cycles of PCR to generate the final double-stranded cDNA library. For quality check, we used the QX200 Droplet Digital PCR EvaGreen Supermix System (Cat. No. 1864034; BioRad, United States) and the High Sensitivity DNA Analysis Kit (Cat. No. 5067-4626; Agilent, United States). Finally, the 12 libraries were pooled in equal ratio and sequenced in one 101-bp single-read run on HiSeq 2500 (Illumina, United States) using one flowcell by Yourgene Bioscience (New Taipei City, Taiwan).

The Illumina raw reads were trimmed at the first position from the 5′-end that has a quality score of <20; reads that are <50-bp after trimming were discarded. The resulting reads were mapped to each genome using BWA v0.7.12 (Li and Durbin, 2009). The mapping results were processed by using SAMtools v1.2 (Li et al., 2009) and BEDTools v2.17.0 (Quinlan and Hall, 2010) to calculate the read counts. NOISeqBio (Tarazona et al., 2015) was used to normalize the read counts and to infer the probability of differential expression. We chose the reads per kilobase per million (RPKM) mapped reads method for normalization; the length correction option was enabled (“lc = 1”) and the counts per million (CPM) method was used for low count filtering with the default cutoff (“filter = 1, cpm = 1”). The criteria for defining differential expression were set to: (1) at least two-fold difference in RPKM values averaged across the three biological replicates, and (2) the probability of differential expression as inferred by NOISeqBio to be at least 0.99. To test the effect of normalization method, we also used the trimmed mean of M (TMM) and the transcripts per kilobase million (TPKM) mapped reads methods. Because both alternatives produced normalized counts with *R*^2^ >0.99 when compared with the RPKM results, we report only the RPKM values.

### Promoter Sequence Analysis

To identify the *cis*-regulatory elements that may be involved in transcriptional control, the 600-bp region upstream of all differentially expressed genes were assigned to four groups (i.e., up- or down-regulated in C58 or 1D1609) for analysis. The program MEME (Bailey et al., 2009) was used for *ab initio* identification of novel motifs. The options “-dna -nmotifs 5” were used to specify the input sequence type and to increase the number of motifs to be identified in each set (only the top one motif is reported under the default setting). The option “-maxw,” which defines the maximum width of motifs, was gradually reduced from 50 (i.e., default) to 15 to test if the results were sensitive to this parameter. In addition to the identification of novel motifs, the upstream regions of all up-regulated genes were checked for the consensus VirG-binding motif RTTDCAWWTGHAAY with up to three mismatches allowed (Cho and Winans, 2005). Those genes that are <50-bp apart and located on the same strand were considered as belonging to the same regulon (e.g., *virB* and *virD*), thus the presence of a motif in the upstream gene was counted as presence for the downstream gene.

## Results and Discussion

### Comparison of Gene Content and Synteny

**FIGURE 1:**
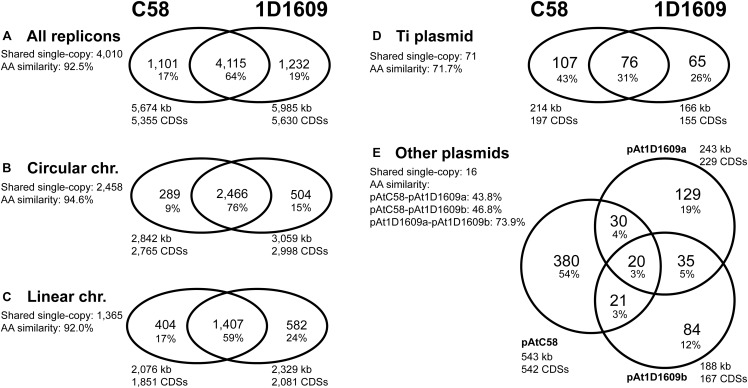
Numbers of shared and strain-specific homologous gene clusters. The percentages of total are labeled in parentheses below gene cluster counts. **(A)** Genome-level comparison; including all replicons. **(B)** Circular chromosome. **(C)** Linear chromosome. **(D)** Ti plasmid. **(E)** Other plasmids. The number of shared single-copy genes in each comparison, as well as the average amino acid similarity of these genes, are provided. Additionally, the replicon sizes and the numbers of coding sequences (CDSs) are labeled.

**FIGURE 2:**
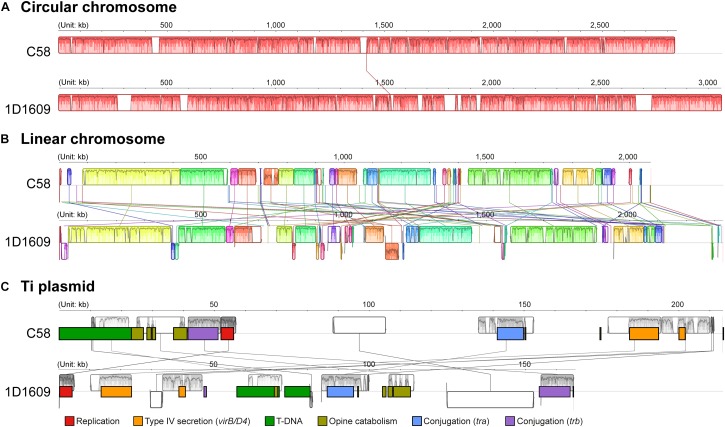
Genome alignment by replicon. **(A)** Circular chromosome; synteny conservation was found across the entire chromosome. **(B)** Linear chromosome; collinear blocks are indicated by colors and linked by lines. **(C)** Ti plasmid; collinear blocks are indicated by gray boxes and linked by lines, notable gene clusters are color-coded.

These two *A. tumefaciens* strains, C58 and 1D1609, share the same chromosomal organization of having one circular chromosome and one linear chromosome, which is conserved within this species complex and unique compared to its Rhizobiaceae relatives (Slater et al., 2009; Wibberg et al., 2011; Slater et al., 2013; Huang et al., 2015, 2017; Cho et al., 2018). For genome-wide comparison, these two strains share ∼4,000 protein-coding genes and these genes have an average amino acid similarity of 92.5% (Figure 1 and Supplementary Table S1). When the replicons were examined individually, the circular chromosomes are more conserved than the linear chromosomes in both gene content and sequence (Figure 1). Consistent with these observations, the genome-level alignments (Figure 2) also revealed that the level of synteny conservation is higher in circular chromosomes than in linear chromosomes.

The two Ti plasmids belong to different opine types, with pTiC58 being the nopaline-type and pTi1D1609 being the octopine-type. We found that these two Ti plasmids share <50% of their protein-coding genes (Figure 1). The shared genes are those related to the Ti plasmid functions (e.g., replication, type IV secretion system, etc.) and are distributed in several small syntenic blocks. However, the global alignment between these two Ti plasmids revealed little synteny conservation (Figure 2). Finally, the three other plasmids found in these two strains, pAtC58 in C58 and pAt1D1609a/pAt1D1609b in 1D1609, all of which are large (i.e., 188–543 kb) *repABC* plasmids, share only 3% of their protein-coding genes (Figure 1). These findings suggested that these three plasmids do not share a common evolutionary origin.

Based on a previous study regarding *Agrobacterium* chromosome evolution (Slater et al., 2009), the dual chromosome organization was derived from intragenomic gene transfer from the ancestral circular chromosome to a large plasmid, followed by linearization of this large plasmid after the divergence of *A. tumefaciens* from its sister lineage that evolved to be *Agrobacterium vitis*. It was hypothesized that this partition of the genome into two chromosomes provided an alternative reservoir for newly acquired DNA, thus reduced the limit that may have been imposed on chromosome size and allowed for continued expansion of gene content. Our results further suggested that the genes located on different replicons may have evolved under different constraints. The circular chromosome appeared to be the core component of *A. tumefaciens* genome and has been under stronger purifying selection than other replicons. In comparison, the linear chromosome harbors more strain-specific genes and the shared genes exhibit a higher level of sequence divergence, which may contribute more to long-term strain-specific adaptation. Finally, the plasmids exhibit the highest level of genetic diversity, both in terms of gene content and sequence variation. Although the large plasmids found in *Agrobacterium* (i.e., pTi and pAt) are generally quite stable and not easily lost in lab culture, these non-essential replicons could be gained or lost rapidly on an evolutionary timescale (as evident in the comparison between these two strains of *A. tumefaciens*). Therefore, plasmids may facilitate short-term adaptation, such as those examples observed for the acquisition of antibiotic resistance (Bennett, 2009).

### Overview of the RNA-Seq Results

For the RNA-Seq experiment, we obtained a total of 316,689,381 Illumina raw reads for the 12 samples (i.e., two strains, three biological replicates, and two conditions). This data set has been deposited in NCBI sequence read archive (SRA) under the accession SRP156105. After quality trimming, we obtained on average ∼21 million reads per sample and ∼97.7% mapping rate (Table 1). The expression levels of all annotated genetic features in these genomes were measured based on the RPKM method and reported in Supplementary Table S2.

**Table 1:** Summary statistics of the RNA-Seq experiment.

Strain	Replicate	Treatment	Raw reads	Filtered reads	Mapped reads	% Mapped
C58	A	Control	19,942,293	15,994,563	15,660,193	97.9
		Induced	14,195,277	11,368,992	11,023,205	97.0
	B	Control	31,861,453	25,354,147	24,852,325	98.0
		Induced	23,660,980	18,928,139	18,445,548	97.5
	C	Control	25,517,439	20,452,423	20,073,945	98.1
		Induced	32,220,286	25,863,961	25,136,728	97.2
1D1609	A	Control	24,405,612	19,563,523	19,085,567	97.6
		Induced	26,151,683	20,900,667	20,451,669	97.9
	B	Control	28,180,612	22,595,680	22,064,805	97.7
		Induced	28,478,759	22,777,150	22,331,056	98.0
	C	Control	27,347,710	21,531,890	21,029,421	97.7
		Induced	34,727,277	27,867,367	27,344,646	98.1

Av.			26,390,782	21,099,875	20,624,926	97.7

Among the mapped reads, 84.1 and 7.0% were mapped to the sense and antisense strand of protein-coding genes, respectively. Reads that were mapped to other annotated non-coding RNA (ncRNA) genes accounted for 7.3%. The remaining reads were mapped to pseudogenized protein-coding genes (0.2%), tRNA genes (0.7%), rRNA genes (0.1%), and intergenic regions (0.6%). This result indicated that our rRNA removal step during the sequencing library preparation was highly effective.

For more detailed analysis, this study focused on the sense strand of protein-coding genes because our sequencing library preparation and data analysis pipeline were optimized for these regions. Although antisense RNAs (asRNAs) were known to be important in the regulation of gene expression (Georg and Hess, 2011), analysis on the global pattern of asRNA expression is difficult due to the biological noise introduced by inefficient transcription control and analytical artifacts caused by expression of adjacent genes on the opposite strand (Nicolas et al., 2012; Raghavan et al., 2012; Lloréns-Rico et al., 2016). For other ncRNAs, although 19 of the 43 annotated features were found to have >0.99 probability of differential expression, none of the these ncRNAs reached the two-fold expression level change cutoff (Supplementary Table S2).

**FIGURE 3:**
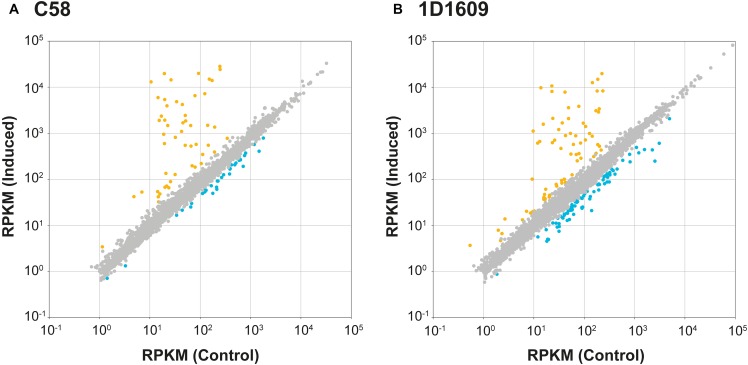
Scatter plots of gene expression levels under the control and the induced condition. **(A)** C58. **(B)** 1D1609. The expression levels are measured in Reads Per Kilobase per Million (RPKM) mapped reads. Genes exhibiting significant differential expressions between the two conditions are highlighted in color (orange: up-regulated by induction; blue: down-regulated by induction); those that do not reach the significance threshold are plotted in gray.

**FIGURE 4:**
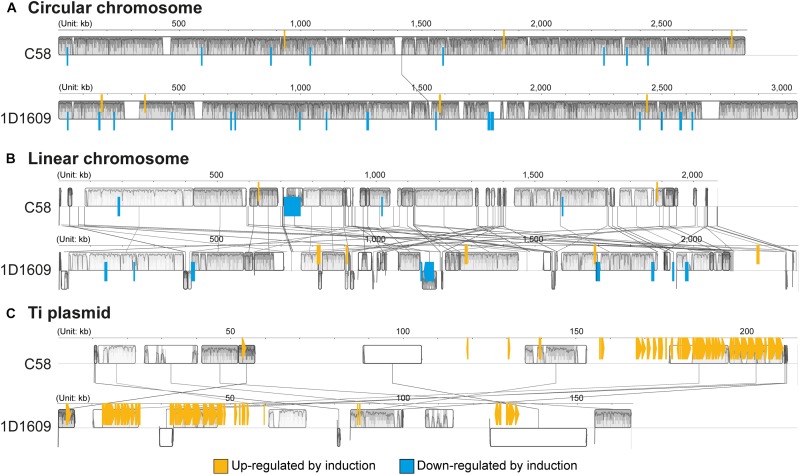
Genomic location of differentially expressed genes. **(A)** Circular chromosome. **(B)** Linear chromosome. **(C)** Ti plasmid. Genes up-regulated by virulence induction are highlighted in orange, genes down-regulated are highlighted in blue.

Among the protein-coding genes, 88/5,355 in C58 and 155/5,630 in 1D1609 satisfied our criteria for differential expression (Figure 3 and Supplementary Table S3). The vast majority of these genes exhibited highly consistent expression levels across biological replicates (Supplementary Figure S1 and Supplementary Table S3). When the differentially expressed genes were classified by their genomic locations, the majority of up-regulated genes are those *vir* regulon genes located on the Ti plasmids (Figure 4, 5A). This observation is consistent with our understanding of *Agrobacterium* biology (Nester, 2015) and confirmed that our acetosyringone treatment (i.e., the induced condition) successfully mimicked the virulence condition. Interestingly, the majority of down-regulated genes are located on the linear and circular chromosomes. This finding suggested that these chromosomal genes may also play a role in *Agrobacterium* virulence. Consistent with this hypothesis, a previous experiment that performed reciprocal exchange of Ti plasmids between C58 and 1D1609 found that the two recombinant strains were both distinct from their parental strains in infectivity profiles (Palumbo et al., 1998). The possible interactions among chromosome- and plasmid-encoded genes remain to be further investigated.

**FIGURE 5:**
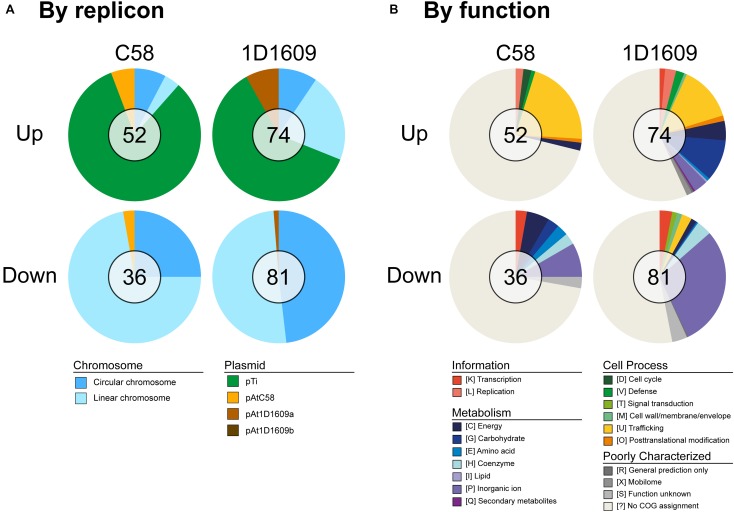
Characteristics of differentially expressed genes. **(A)** By replicon. **(B)** By function. The total number of genes in each group is labeled in the center of each pie chart.

When the differentially expressed genes were classified by their functional categories, we found that more than half of these genes lack specific annotation (Figure 5B). These findings provided a targeted list for future functional characterization by molecular genetic approaches. Among those with more specific annotation, the type IV secretion system (T4SS) and *vir* genes on the Ti plasmid represented the majority of up-regulated genes, which is consistent with our expectation of virulence induction. Intriguingly, the comparison between these two strains revealed that 1D1609 has several carbohydrate metabolism genes that were up-regulated, while the homologs in C58 did not show significant change in expression. These include those genes for phosphonate transport system (*phnC/D*) and cytochrome oxidase (*fixO/P/Q*) on the circular chromosome, as well as a gluconolactonase gene and its adjacent ribose transporter genes located on the linear chromosome (*rbsA/B/C*). Among the down-regulated genes with specific functional assignments, those involved in zinc/iron ion transport were the most abundant groups in both strains.

The between-strain comparison that considered homology (Table 2) revealed more complex patterns than those observed by classifying genes based on genomic location (Figure 4, 5A) or functional assignment (Figure 5B). For example, only 23 homologous genes were up-regulated in both strains, which account for <50% of the up-regulated genes in each strain. The remaining ones either lack homologous genes in the other strain or the homologous genes did not reach the significance threshold of differential expression (e.g., those aforementioned carbohydrate metabolism genes). A similar pattern was observed among the down-regulated genes as well. These differences in gene regulation, as well as in gene content (Figure 1), may explain the phenotypic differences between these strains with regard to infection efficiency or host-specificity (Palumbo et al., 1998; Hwang et al., 2013).

**Table 2:** Numbers of differentially expressed genes.

C58	1D1609	Count
Up	Up	23
Up	Down	1
Up	Not significant	10
Up	Absent	18
Down	Up	0
Down	Down	20/18^a^
Down	Not significant	11
Down	Absent	5
Not significant	Up	25
Not significant	Down	26
Absent	Up	26
Absent	Down	36

*^a^One homologs gene cluster (i.e., cluster #20; siderophore biosynthesis protein) contains three homologs in C58 and one homolog in 1D1609, such that the gene counts differ by two between these two strains.*

Our examination of the upstream regions of these differentially expressed genes confirmed that most of the up-regulated genes (i.e., 44/52 in C58 and 61/74 in 1D1609) are associated with the consensus VirG-binding motif (Cho and Winans, 2005; Supplementary Table S4). In addition to the *vir* genes located on the Ti plasmids, these genes included those without a clear link to *vir* regulons (e.g., multiple genes annotated as hypothetical proteins; C58: Atu6137, Atu6155, Atu6157, Atu6160, Atu6162, and Atu6165; 1D1609: At1D1609_51170, At1D1609_51280, At1D1609_51400, At1D1609_51420, At1D1609_51520, At1D1609_51770, and At1D1609_52200) or located on other replicons (e.g., C58: Atu2782-Atu2783 on the circular chromosome and Atu3578 on the linear chromosome; 1D1609: At1D1609_01540-At1D1609_01550 on the circular chromosome and At1D1609_41520-At1D1609_41560 on the linear chromosome). These findings further supported the importance of VirG in regulating *Agrobacterium* virulence and suggested that VirG may also regulate genes located outside of Ti plasmids.

Unfortunately, no strong candidate for novel *cis*-regulatory element was identified in the *ab initio* motif searches. The motifs identified in each set of differentially expressed genes were highly sensitive to the search parameters used. Longer motifs tended to be preferentially identified when the settings allowed. However, as the setting for maximum width reduced, the shorter motifs identified under such settings often did not belong to sub-strings of the longer motifs. The only motif that could be recovered robustly under various settings was the consensus VirG-binding motif (Cho and Winans, 2005). A possible explanation for this result is that the down-regulated genes belong to multiple regulatory modules, thus the statistical signals for the motifs associated with each module are difficult to detect.

### Notable Genes With Differential Expression Patterns

The *vir* regulon genes are known to be crucial for *Agrobacterium* virulence against their plant hosts (Nester, 2015). While almost all of the *vir* genes are located on the Ti plasmid and were highly up-regulated by acetosyringone induction in both strains (Figure 2C, 4C), there are several exceptions to this general observation. First, *virA* was up-regulated by >8-fold in C58 but only ∼18% in 1D1609 (Supplementary Tables S2, S3). The difference was attributed to the extent of up-regulation, as well as a ∼2-fold difference in basal expression (i.e., expression levels based on RPKM Av. ± Std. Dev.; C58: control = 66 ± 3, induced = 526 ± 67; 1D1609: control = 120 ± 4, induced = 142 ± 7). Moreover, while *virA* is located on the Ti plasmid in C58 and other characterized *Agrobacterium* strains (e.g., Ach5), this gene is located on the accessary plasmid pAt1D1609a in 1D1609. Related to this, two other *vir* genes (i.e., *virK* and *virH2*; neither is essential for tumor formation) in 1D1609 are also found on pAt1D1609a instead of pTi1D1609. Nevertheless, both *virK* and *virH2* in 1D1609 were highly induced by acetosyringone (∼3- and ∼39-fold up-regulation, respectively). In contrast to the pattern observed for *virA*, *virG* was highly up-regulated in 1D1609 (∼18-fold) but less so in C58 (∼41%). Intriguingly, the basal expression level of *virG* in C58 was ∼5-fold higher (i.e., expression levels based on RPKM Av. ± Std. Dev.; C58: control = 899 ± 31, induced = 1,270 ± 93; 1D1609: control = 171 ± 12, induced = 3,107 ± 219). Given the important role of the VirA/VirG two component system in *vir* regulon activation, these differences between C58 and 1D1609 warrant further investigation.

One other gene belonging to the *vir* regulon, *tzs*, was up-regulated by >200-fold in C58 and absent in 1D1609. This gene encodes a *trans*-zeatin secretion protein and is specific to nopaline strains while absent in octopine strains (Akiyoshi et al., 1987). Although *tzs* is not essential for transformation, its role in stimulating plant cell division could improve transformation efficiency. More importantly, the transformation efficiency of an octopine strain LBA4404 could be improved by introducing the *tzs* from pTiC58 (Zhan et al., 1990). It remains to be tested if the introduction of *tzs* could improve transformation or alter host range in 1D1609 and other *Agrobacterium* strains lacking this gene.

Among all differentially expressed genes, one stood out as exhibiting opposite patterns between these two strains (i.e., up-regulated by >7-fold in C58 and down-regulated by 2-fold in 1D1609). This gene is annotated as a dehydrogenase and is located on the circular chromosome in both genomes (locus tags: Atu0946 in C58 and 1D1609_09900 in 1D1609). The protein sequence contains a functional domain for NADP-dependent saccharopine dehydrogenase (accession: COG1748), so it is possibly involved in the alpha-aminoadipate pathway of lysine biosynthesis.

Finally, those genes that exhibited differential expression in 1D1609 and lack homolog in C58 could be important for our understanding on the genetic differentiations in *Agrobacterium*. For those with more informative annotation, we found one gene cluster (locus tags: At1D1609_51400-At1D1609_51430) that was up-regulated and contains a cold-shock protein (*csp*). Because *csp* could be a pathogen-associated molecular pattern (PAMP) recognized by plant immune system (Saur et al., 2016), the up-regulation of this gene cluster could affect the infection process. Another up-regulated gene cluster (locus tags: At1D1609_52200-At1D1609_52230) contains a fructoselysine 6-phosphate deglycase (*frlB*) and a 3-oxoacyl-[acyl-carrier-protein] reductase (*fabG*). These enzymes may participate in the metabolism of amino acids, carbohydrates, and fatty acids. For the down-regulated gene clusters, several encode iron transporters (i.e., At1D1609_12720-At1D1609_12760, At1D1609_45370-At1D1609_45410, At1D1609_46920-At1D1609_46950, and At1D1609_47720-At1D1609_47730), suggesting that iron homeostasis could be important. In addition, one large gene cluster on the circular chromosome contains 12 consecutive genes encoding hypothetical proteins (locus tags: At1D1609_17610-At1D1609_17720), all of which were down-regulated. The function and physiological significance of these 12 genes remained unclear.

## Conclusion

In this work, we compared the genomes and transcriptomes of two wild-type *Agrobacterium* strains that differ in host range and infection efficiency. Comparative genomics between these two strains revealed a high level of gene content variation, particularly in their plasmids. Furthermore, comparative transcriptomics that examined the expression response to virulence induction identified ∼100–150 differentially expressed genes in each strain. Notably, many homologous genes exhibited inconsistent patterns of expression regulation between these two strains, indicating that the divergence in gene regulation may also explain their phenotypic differences. Finally, a short list of strain-specific genes that were highly up- or down-regulated by virulence induction yet lacked functional annotation were identified (Supplementary Table S3). These genes are promising candidates for future functional study to better understand *Agrobacterium* virulence. Taken together, this study provided a strong foundation for future efforts to improve the efficiencies or host range of *Agrobacterium*-mediated transformation, thus contributes to basic biological research and biotechnology applications.

## Data Availability

The Illumina raw reads have been deposited at the NCBI SRA under the accession number SRP156105.

## Author Contributions

E-ML and C-HK designed the experiments. MH, S-TC, M-JF, A-PC, and S-JC performed the experiments. MH, S-TC, E-ML, and C-HK analyzed the data. MH, S-JC, E-ML, and C-HK wrote the manuscript. C-HK acquired the funding and supervised the project.

## Conflict of Interest Statement

The authors declare that the research was conducted in the absence of any commercial or financial relationships that could be construed as a potential conflict of interest.

## Supplementary Material

FIGURE S1 Heatmap visualization of the gene expression levels. Each row represents one of the differentially expressed genes, the expression levels are normalized across samples and converted into *Z*-scores for visualization (above average: orange; below average: blue).

TABLE S1 Homologous gene clusters between C58 and 1D1609. Each row in the table lists one protein-coding gene; the cluster id (as assigned by OrthoMCL) and other information (e.g., genomic location and annotation) are included. Genes sharing the same cluster id are considered as homologs. Genes without any identifiable homolog were assigned to their own clusters with unique ids (i.e., singletons).

TABLE S2 Transcriptome mapping results. For each gene, the expression level observed from each of the three biological replicates (i.e., A–C), the mean expression levels under the two conditions, the probability that the gene was differentially expressed, and the log_2_ fold-change in the mean expression levels are reported.

TABLE S3 Lists of differentially expressed genes. Four separate sheets are included for listing the genes by genome or by the directionality of differential expression. The cluster id for each gene refers to the results of homologous gene cluster identification; genes sharing the same cluster id are considered as homologs.

TABLE S4 Association between VirG-binding motifs and up-regulated genes. Four separate sheets are included; each strain has one sheet for a summary report and one sheet for detailed information regarding the locations and the exact sequences of motifs.
