# Supplementary figures and images for: Differentiations in Gene Content and Expression Response to Virulence Induction Between Two Agrobacterium Strains
Source: Front Microbiol. 2019 Jul 9;10:1554. doi: 10.3389/fmicb.2019.01554 (PMC6629968; doi:10.3389/fmicb.2019.01554)

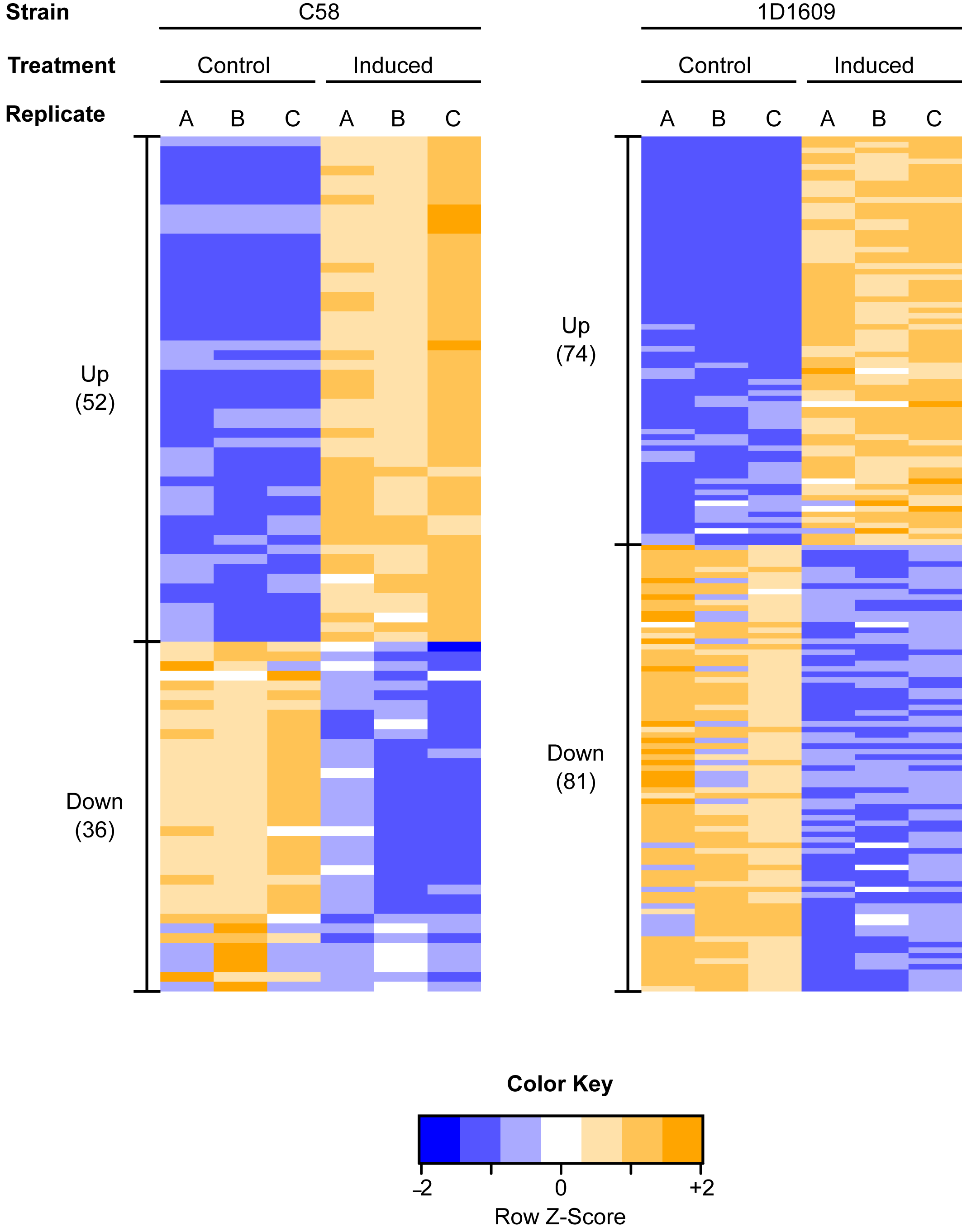

Supplement: FIGURE S1 — Heatmap visualization of the gene expression levels. Each row represents one of the differentially expressed genes, the expression levels are normalized across samples and converted into Z-scores for visualization (above average: orange; below average: blue). [file Image_1.TIF]
